# Pseudo-precocious puberty in a 3-year old girl

**DOI:** 10.1186/1687-9856-2013-S1-P78

**Published:** 2013-10-03

**Authors:** Catherine Anne G Pangilinan-Vazquez, Sioksoan C Cua, Lorna R Abad, Carmelita F Domingo, Sylvia C Estrada, Caridad M Santos

**Affiliations:** 1 Section of Pediatric Endocrinology, University of the Philippines – Philippine General Hospital, Manila, Philippines

## 

Sertoli Cell Tumor is a rare sex cord tumor, comprising less than 5% of all sex cord tumors. It usually occurs in women of reproductive age but a few can also occur during early childhood. The usual manifestation among children is isosexual pseudoprecocity.

This is a case of a three-year-old girl who came in with a chief complaint of vaginal bleeding. She was born term to a G2P1 mother with no perinatal complications noted. At two years of age, she was noted to have gradual bilateral breast enlargement. There was no breast discharge noted. At two years and four months of age, she started to have vaginal bleeding which the mother claimed to occur on a “monthly” interval lasting for five to seven days. There was no history of trauma or any accompanying urinary symptoms. Initial consult was done in a local hospital where she was also noted to have enlarged head. Cranial CT Scan done was normal. Assessment made was precocious puberty. The patient was subsequently referred to our institution for further evaluation. On physical examination, pertinent findings include increased head circumference (53.5 cm, z-score > 3 S.D.), weight (16 kg, z-score >0) and height (100 cm, z-score = 0) within normal for age, Tanner Stage 3 for breast and Stage 1 for pubic hair. The rest of the physical exam findings were essentially normal. Bone age was appropriate for chronologic age. Hormonal work-up were as follows: FSH: 0.27 (NV:0.27-3.39 IU/L), LH: 0.22 (NV: 0.03-0.55 IU/L), Estradiol: 234 (NV:<10pg/mL), Prolactin: 783 (NV:80-500 microIU/mL), TSH: 9.4 (0.3-3.8 mIU/L), FT4: 22.4 (11-24 pmol/L). Pelvic ultrasound revealed upper borderline-sized uterus with slightly thickened round complex mass in the left adnexal region, to consider an ovarian neoplasm. Alpha-feto Protein, B-HCG, CA-125 tumor marker levels were all within normal limits. The patient subsequently underwent Exploratory Laparotomy with Left Salphingo-oophorectomy. Intra-operatively, the left ovary was noted to be converted to a 5cm x 5cm x 3cm solid mass with smooth surface and intact capsule. The right fallopian tube and ovary appeared normal. Histopathologic studies were consistent with Sertoli Cell Tumor of the left ovary, positive for Inhibin and Calretinin immunostaining. The vaginal bleeding and progressive breast enlargement ceased post-operatively. The estradiol and prolactin levels dropped (Estradiol: 234 to 37.3pg/mL and Prolactin: 783 to 304 microIU/mL) one month after the surgery.

